# The Host Response to Coccidioidomycosis

**DOI:** 10.3390/jof10030173

**Published:** 2024-02-25

**Authors:** Theo N. Kirkland, Chiung-Yu Hung, Lisa F. Shubitz, Sinem Beyhan, Joshua Fierer

**Affiliations:** 1 Department of Medicine, Division of Infectious Disease, San Diego School of Medicine, University of California, San Diego, CA 92093, USA; sbeyhan@health.ucsd.edu (S.B.); jfierer@health.ucsd.edu (J.F.); 2 Department of Pathology, San Diego School of Medicine, University of California, San Diego, CA 92093, USA; 3 South Texas Center for Emerging Infectious Disease, Department of Molecular Microbiology and Immunology, University of Texas at San Antonio, San Antonio, TX 78249, USA; chiungyu.hung@utsa.edu; 4 Valley Fever Center for Excellence, University of Arizona, Tucson, AZ 85724, USA; lfshubit@arizona.edu; 5 Department of Infectious Diseases, J. Craig Venter Institute, La Jolla, CA 92037, USA; 6 Infectious Diseases Section, VA Healthcare San Diego, San Diego, CA 92161, USA

**Keywords:** coccidioidomycosis, immunity, innate immunity, macrophages, dendritic cells, genetics, acquired immunity, vaccines

## Abstract

Coccidioidomycosis is an important fungal disease that is found in many desert regions of the western hemisphere. The inhaled organisms are highly pathogenic, but only half of infected, immunologically intact people develop symptomatic pneumonia; most symptomatic infections resolve spontaneously, although some resolve very slowly. Furthermore, second infections are very rare and natural immunity after infection is robust. Therefore, the host response to this organism is very effective at resolving the infection in most cases and immunizing to prevent second infections. People who are immunocompromised are much more likely to develop disseminated infection. This is a comprehensive review of the innate and acquired immune responses to *Coccidioides* spp., the genetics of resistance to severe infection, and the search for an effective vaccine.

## 1. Introduction

*Coccidioides* spp. are highly pathogenic organisms but only 50% of immunocompetent hosts develop symptomatic disease. A large majority of pulmonary infections are self-limited and often go undiagnosed. Thus, the human immune system is usually able to control the infection with either no residual evidence of prior infection or a solitary pulmonary nodule [1,2,3]. For this reason, evaluating mammalian immunity is critical for an understanding of the host–pathogen relationship. *Coccidioides* species are dimorphic fungi. Their life cycle consists of a saprobic phase characterized by mycelia that produce enterothallic arthroconidia and a parasitic phase characterized by endosporulating spherules. The parasitic cycle of *Coccidioides* is unique amongst the medically important fungi and the ability to form spherules from arthroconidia is required for pathogenicity. Fundamental research on their genetic, morphology, ecology, virulence factors, and pathogenic mechanisms has recently been reviewed [4,5]. Here, we discuss immune responses to coccidioidomycosis in animal models, experiments using human cells in vitro*,* and clinical observations in humans.

## 2. Innate Immunity

There are many reviews of innate immunity in coccidioidomycosis [2,6,7,8,9]. An especially informative and complete review of this topic has recently been published [10]. The innate immune response to infectious organisms plays a key role in slowing the course of the infection while the host is generating an antigen-specific immune response, and the innate response influences the type of adaptive immune response that is made by virtue of the cytokines and chemokines produced by innate immune cells [11]. Many factors are involved in promoting a protective immune response, and the following is our attempt to focus on those most successfully investigated in the laboratory.

### 2.1. Local Factors in the Lung

Pulmonary epithelial cells interact with the arthroconidia very early in the course of infection. There have been some fascinating hypotheses about the importance of this interaction, but no data have been published [12]. Recently, Hsu reported that mutations in Duox1, a lung epithelial cell oxidase that generates H_2_O_2_, were more common in patients with disseminated coccidioidomycosis than in the general population, suggesting that this enzyme plays a role in innate resistance to coccidioidomycosis [13]. Whether arthroconidia or round cells are killed by physiological concentrations of hydrogen peroxide is not known, but there is some evidence that they are relatively resistant to oxidative stress [14]. Pulmonary surfactant is also an early host fluid to encounter arthroconidia in the lung. It has been reported that mice challenged with *C. posadasii* arthroconidia had dramatically lower concentrations of SP-A and SP-D in their bronchoalveolar fluid (BALF) compared to controls 10 days after challenge [15]; immunization of mice before the challenge prevented this effect. It is not clear whether the alterations in surfactants occur early in infection or are a consequence of the development of pneumonia. The roles of epithelial-associated myeloid cells, such as NK and iNKT cells, and epithelial-associated dendritic cells in innate resistance to pulmonary coccidioidomycosis, are unknown.

### 2.2. Polymorphonuclear Leukocytes

**Figure 1:**
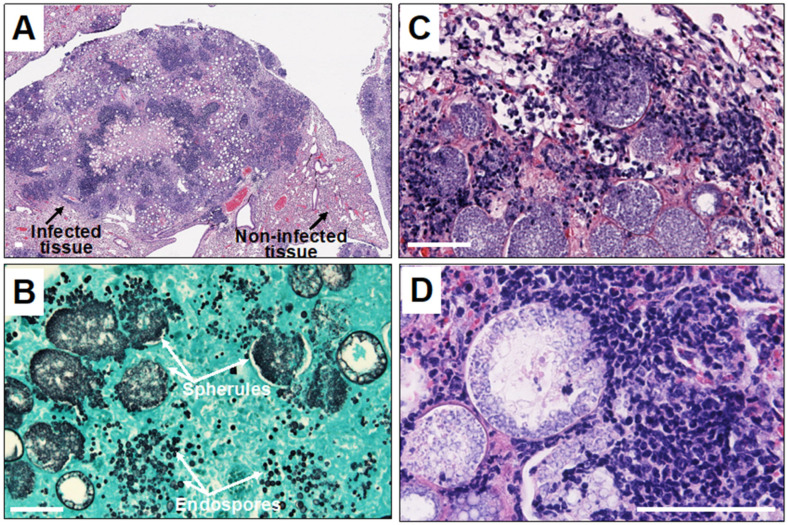
Histopathology of *Coccidioides* infection in mice. Legend: (**A**) microscopic image of *Coccidioides*-infected mouse lung tissue showing disorganized pyogranuloma. Empty circles are large spherules with central vacuoles in the infected tissue; (**B**) large spherules and tiny endospores in the infected tissue stained with Gomori-methenamine silver; and (**C**,**D**) microscopic images showing infiltration of neutrophils and other granulocytes toward endosporulating spherules. The white scale bar represents 100 µm.

Polymorphonuclear leukocytes (PMN) are cells that act early in the course of infection, phagocytosing and inhibiting the growth of arthroconidia to some degree [16]. There is ample histologic evidence that collections of PMNs occur in pathologic lesions, although the original arthroconidia at this point in infection have either died or converted to spherules (Figure 1).

Chemotaxis is required for PMN to move from the circulation to the organism in tissues. In chemotactic assays using mycelial or spherule culture filtrate, complement was found to be required for the chemotactic response [16]. A recent study using very sophisticated imaging techniques found that human PMN recognized spherules and endospores over a distance of several micrometers in the presence of serum [17]. The PMN then phagocytosed both spherules and endospores in the presence of human serum containing active complement.

Human PMNs inhibit chitin synthesis by arthroconidia in vitro, especially in the presence of complement [18]. This inhibition lasted more than six, but less than 24 hours. PMNs from a patient with chronic granulomatous disease did not inhibit chitin synthesis. However, PMN from normal donors did not kill *Coccidioides* spp. over the first 18 h of co-incubation, despite phagocytosis of the arthroconidia and degranulation of the PMNs. Inhibition of chitin synthesis by PMNs or H_2_O_2_ was also observed in small (young) spherules but not in large (mature) spherules [19]. Another study found that both young and mature spherules were phagocytosed very poorly by PMN’s compared to arthroconidia, spherule initials, or endospores [20]. Very few organisms were killed, although there were some differences between the four clinical isolates that were studied. In most cases, phagocytosed endospores did not elicit degranulation of the PMNs, which is consistent with the minimal killing observed. Segal reported that a variety of cationic peptides were able to inhibit the growth of arthroconidia [21]. Studies of cationic peptides with spherules and endospores have not been reported. 

Studies in IL-8 Receptor 2 knockout (IL-8R2^−/−^, also known as CXCR2^−/−^) mice on a BALB/c background suggest that PMN’s may not play a protective role in experimental mouse infection [22]. Although mice do not produce IL-8, they do make the chemotactic factors MIP-2 and KC, which are ligands for this receptor in mice. Normal mouse macrophages stimulated with spherules make MIP-2. As expected, fewer PMN’s were found in the lungs of *C. immitis*-infected IL-8R2^−/−^ mice compared to BALB/c controls. Unexpectedly, the IL-8R2^−/−^ mice had 10-fold lower fungal quantitative culture values (CFU) in their lungs and spleens 14 days after infection compared to the controls. Survival studies were not conducted.

To evaluate the molecular mechanisms behind increased resistance to this infection, RNA-seq studies of mouse genes in the infected lungs were conducted. Upregulated genes in control animals were enriched for gene ontology categories related to leukocyte activation, such as inflammatory response, leukocyte migration, and neutrophil degranulation, consistent with the higher numbers of neutrophils in their lungs. Expression of IL-17, 1L-12, and interferon-γ (IFN-γ) genes, which are protective cytokines, were upregulated in the IL-8R2^−/−^ mice. In contrast, IL-1β, TNF-α, IL-6, and the inhibitory cytokine IL-10 were more upregulated in control lungs [22]. The difference in cytokine profiles provide a rationale for the decreased fungal burden in IL-8R2 KO mice.

In contrast to those results, depletion of PMN by treatment with a monoclonal antibody did not change the severity of infection (as measured by CFU in lungs and spleens) in C57BL/6 mice [23]. The discrepancy between that result and Carlin’s results could be due to the difference in methods for eliminating the PMN response, the mouse strains used, and/or the more severe disease caused by the *C. posadasii* C735 infection compared to the less pathogenic *C. immitis* RS organism.

### 2.3. Monocytes/Macrophages and Dendritic Cells

The biology of monocytes, macrophages, and dendritic cells is very complex and play variety of roles in innate immunity with overlapping functions. It is important to remember that, while macrophages play an important role in the control of all types of infections, monocytes mature into different types that differ in function [24,25]. Dendritic cells are also a complex group of cells involved in sensing infection and, producing cytokines and chemokines that shape the acquired immune response, and they are the most efficient antigen presenting cells to T-lymphocytes [26].

A few older studies have reported that activation of mouse peritoneal macrophages by immune spleen cells resulted in increased phagolysosome fusion and the killing of arthroconidia and endospores [27]. A subsequent study found that peritoneal macrophages incubated with supernatants of antigen-stimulated *Coccidioides*-immune spleen cells also fused the phagolysosome after phagocytosis of arthroconidia. The addition of these supernatants to macrophages increased the killing of arthroconidia [28]. To our knowledge, these observations have not been reproduced by others.

A study about the interaction of arthroconidia with C57Bl/6 mouse macrophages and dendritic cells was recently published [29]. The avirulent *C. posadasii cts2/cts3* deletion mutant was used to evaluate phagocytosis of arthroconidia by mouse macrophages and dendritic cells, a mouse macrophage cell line, and a rat pulmonary macrophage cell line. None of these cell types phagocytosed arthroconidia efficiently; only about 10% of the cells phagocytosed arthroconidia. Incubation of mouse bone-marrow-derived monocytes with arthroconidia resulted in differentiation into M0 macrophages (potentially immunosuppressive), but not into M1 (inflammatory) or M2 (anti-inflammatory) macrophage phenotypes, as characterized by cell surface markers. Incubation of dendritic cells with arthroconidia caused little cellular activation and was biased to the DC1 phenotype (which activates CD4 T-cells poorly) [26]. It would be informative to conduct these experiments with spherules as well as arthroconidia, and with other *Coccidioides* spp. and inbred mouse strains.

### 2.4. Cellular Receptors and Pathways for the Innate Immune Response

#### 2.4.1. C-Type Lectin Receptors

Dectin-1, (the β-glucan receptor that is expressed on myeloid cells and some lymphocytes) [30] was first shown to be required for a mouse macrophage cell line to produce pro-inflammatory cytokines when stimulated with inactivated spherules or β-glucan isolated from spherules. Cells that did not express Dectin-1 were unresponsive to those stimuli and an anti-Dectin-1 monoclonal antibody substantially reduced the pro-inflammatory cytokine response of mouse peritoneal elicited macrophages to spherules. Therefore, Dectin-1 appears to be an important receptor for the pro-inflammatory cytokine response of murine peritoneal macrophages to spherules. Furthermore, elicited peritoneal macrophages from C57BL/6 Dectin-1^−/−^ mice made much less TNF-α, IL-6, and MIP-2 in response to spherules than the peritoneal macrophages from control C57BL/6 mice [31]. However, the response of bone-marrow-derived dendritic cells (BMDC) to spherules was somewhat different. The IL-6, IL-10, and granulocyte-macrophage colony-stimulating factor (GM-CSF) responses to spherules were mostly dependent on Dectin-1, but IL-1 and TNF-α production was independent of this receptor. The course of infection was also influenced by Dectin-1. C57BL/6 *Clec7a*^−/−^ mice (lacking Dectin-1) had significantly more organisms in their lungs and substantially more organisms in their spleens compared to the controls, suggesting that the activation of Dectin-1 reduces dissemination from the lungs [32]. In addition, bronchial alveolar lung fluid (BALF) from Dectin-1^−/−^ mice obtained after intra-nasal infection contained lower concentrations of protective T_H_17 and T_H_1 cytokines than BALF from infected control mice. T_H_17 cytokines are required for the successful immunization of mice to *Coccidioides* spp. with an attenuated live vaccine [33], although the role of T_H_17 cytokines in human coccidioidomycosis is not yet established.

Murine susceptibility to experimental coccidioidomycosis is greatly influenced by the genetic background of the animal: C57BL/6 mice are relatively susceptible to infection and DBA/2 are relatively resistant [34]. The two strains of mice had the same amount of Dectin-1 in macrophages, but the Dectin-1 transcript was about 100 bp shorter in the C57BL/6 mice than in the DBA/2 mice. The truncated Dectin-1 lacked the extracellular stalk, which was the result of alternative splicing of *clec7a* by C57BL/6 mice that eliminated the expression of exon 3 [35]. C57BL/6 x DBA/2 recombinant inbred (BXD RI) mice with the truncated form of Dectin-1 were more susceptible to *C. immitis* infection than the RI mice that expressed the full-length protein [31]. A cell line transfected with the truncated C57BL/6 isoform of Dectin-1 made half as much TNF-α as those transfected with full-length Dectin-1 when stimulated with killed spherules. This is the opposite of how the two splice variants affect macrophage responses to *C. albicans* [35]; therefore, these two organisms elicit different responses to the truncated protein. In contrast to these studies, Campuzano et al. did not find that Dectin-1^−/−^ C57BL/6 mice were more susceptible to a primary infection with *C. posadasii* C735 than C57BL/6 controls, although vaccination of the mutant mice with a live-attenuated vaccine (ΔT strain) did not protect Dectin-1^−/−^ mice as well as the controls [33]. This may be because the infection in unvaccinated C57BL/6 mice was so severe that it was not possible to detect the immune defect caused by the absence of Dectin-1 [33]. However, vaccination of Dectin-1^−/−^ mice with a different live-attenuated vaccine (Δ*cps1*) was relatively protective against infection with *C. posadasii* Silveira [36]; so the role of Dectin-1 in vaccine-induced immunity is unclear.

The roles of murine Dectin-2 and the mannose receptor (MR) on cytokine secretion in vitro and resistance to infection in vivo have also been studied. BMDC from C57Bl/6 MR^−/−^ mice make less pro-inflammatory cytokines in vitro in response to spherules compared to controls, even though the MR receptor is not a signaling receptor [37]. However, MR^−/−^ mice are not more susceptible to *C. immitis* infection [37]. Furthermore, the mannose receptor plays a role in the recognition of *C. posadasii* spherules by human DCs, since spherule binding and cytokine production can be inhibited by mannan [38,39]. The discrepancy between the effect of the MR on vitro response and the course of infection is unexplained.

Peritoneal macrophages from Dectin-2^−/−^ mice made poor pro-inflammatory cytokine responses to spherules in vitro. Dendritic cells (BMDC) from Dectin-2^−/−^ mice made identical protective cytokine responses in vitro to spherules, but lower amounts of IL-10. The Dectin-2^−/−^ deletion had no effect on susceptibility to *C. immitis* infection [37]. Mice lacking both MR and Dectin-2 were not more susceptible to infection, indicating that these receptors did not play a critical role in host defense to coccidioidomycosis in mice, despite the role they play in vitro.

Dectin-2 does play a role in the acquired immune response to *Coccidioides* spp. [33,40]. Immunization of Dectin-2^−/−^ mice with a live, attenuated mutant led to lower levels of IL-17 as well as IFN-γ. Immunized Dectin-2^−/−^ mice were more susceptible to infection than immunized controls. Vaccine protection was dependent on the production of T_H_17 cells, and very few T_H_17 cells were present in the lungs of vaccinated Dectin-2 KO mice after infection. CARD-9 is an intracellular signal transduction adaptor molecule that is downstream of Dectin-1 and Dectin-2. CARD-9^−/−^ mice were not protected by vaccination.

Recently, Hsu et al. confirmed the importance of Dectin-1 in humans by demonstrating that a mutation in *Clec7a* that eliminates the C terminal β-glucan binding site was more prevalent in patients with disseminated coccidioidomycosis than in the general population, and that their monocyte-derived macrophages did not make TNFα when stimulated with curdlan (a Dectin-1 agonist) (see Section 2.6) [13]. Therefore, it seems that Dectin-1 is important for innate immunity in people as well as mice. The role of Dectin in human coccidioidomycosis deserves further study.

#### 2.4.2. Toll-Like Receptors (TLR)

Elicited peritoneal macrophages from C57Bl/6 TLR-2^−/−^ mice made a very poor pro-inflammatory cytokine response to spherules in vitro compared to macrophages from control mice [41]. However, BMDC from both MyD88^−/−^ and MyD88/TRIF^−/−^ mice made as much TNF-α, IL-6 and IL-10 in response to spherules as the control C57BL/6 mice [42]. Macrophages from TLR-4^−/−^ mice made an identical response to spherules, compared to control mice, suggesting that TLR-4 is not an important receptor for the macrophage response to spherules. This observation is consistent with a previous study of genetically determined resistance to *C. immitis* in mice, in which a TLR-4 null mutation was found to have no effect on resistance to infection [34]. There are no data about the role of TLR on pulmonary macrophages in the response to spherules.

Despite the observation that MyD88 did not affect the cytokine response to spherules by BMDC in vitro, MyD88^−/−^ and MyD88/TRIF^−/−^ mice were more susceptible than controls to *C. immitis* infection. However, neither TLR-2^−/−^ nor TLR-4^−/−^ mice were more susceptible to infection. Since MyD88 is a signal-transducing molecule for both the IL-18 receptor and the IL-1 receptor, the role of each of these receptors on resistance to infection was studied. IL-18R^−/−^ mice were no more susceptible to infection than control mice, but IL-1 receptor^−/−^ mice were much more likely to develop disseminated disease. IL-1RA, part of the IL-1 family of cytokines, was present at a higher concentration than any other measured cytokine in the BALF of infected C57BL/6 mice, further evidence of the importance of the IL-1 family in pathogenesis [22]. These experiments suggest that signaling by IL-1 may play a critical role in innate immunity to coccidioidomycosis in mice. The IL-1 receptor and MyD88 have both been shown to be important for vaccine-induced immunity in mice [23]. The role of IL-1 in the human innate response to *Coccidioides* has not been studied.

#### 2.4.3. In Vivo Studies of Murine Innate Immunity

Barker and colleagues have evaluated the response to pulmonary infection with 10^5^ arthroconidia (an extremely high inoculum) using three different strains (two *C. immitis* and one *C. posadasii*) at days 1–5 after challenge [43]. The strains differed in pathogenicity: the *C. posadasii* strain Silveira was the most pathogenic, whereas the *C. immitis* strain 2006 was least pathogenic, and *C. immitis* R.S. was intermediate. *Coccidioides* spp. RNA could be detected in the lungs of mice infected with all three isolates by day 5 and the leukocyte count was also elevated by that time. The cytokine responses as measured by qRT-PCR of lung tissue showed that IL-1, IL-1R, IL-10, IL-17RA, and the IFN-γ receptor genes were all upregulated in response to all three challenges by day 5 after infection, whereas the other measured cytokines showed less consistent responses. The contents of BALFs were also analyzed by proteomic techniques: 38% (138/366) of the detected mouse proteins were found in mice challenged with all three isolates and 12–42 proteins were unique to each of the three challenges. Most of the detected proteins were not cytokines. One conclusion of this study is that there are differences in the host responses to the three challenge strains, as measured by cytokine production and the proteomics of host proteins.

Innate resistance to subcutaneous infection in mice has also been studied [44]. Subcutaneous inoculation of the organism is an unusual but well-known route of infection in human beings who are injured in desert soil. The course of this type of infection in wildtype mice was resolution of the infection over three months after inoculation. However, CARD-9^−/−^ and MyD88^−/−^ mutant mice in theC57BL/6 background died after the challenge, suggesting the importance of both TLR and C-type lectin signaling in this route of infection. Although the lesions contained many PMNs, PMN depletion with the antibody did not affect the fungal burden. IFN-γ, but not IL-17 was required for resistance to subcutaneous infection. In addition, the fungal burden was significantly higher in iNOS^−/−^ mice than in the controls. Therefore, it seems that the factors determining innate resistance to subcutaneous infection are significantly different than those important for resisting pulmonary challenges.

Another paper also compared the cytokine response in BALF from mice infected with *C. immitis* RS or *C. posadasii* Silveira [45]. The two fungal strains studied elicited different cytokine profiles 10 days after infection, although there was a good deal of variation within each group. In addition, the volatile organic compound profiles in the BALF were found to correlate with cytokine profiles. Whether the mouse or the fungus produces these volatile organic compounds is unknown.

### 2.5. Genetically Determined Resistance to Infection and Innate Immunity in Mice

The initial studies of genetic differences in susceptibility to infection in mice were conducted using intraperitoneal (I.P.) infection with the *C. immitis* RS as a model [34]. In this model, mice develop intra-peritoneal granulomas and infection spread to lungs and other organs. There was a dramatic difference between susceptibility in strains; C57BL/6 and BALB/c mice were very sensitive to infection and DBA/2 mice were relatively resistant. Resistance to infection was not determined by the major histocompatibility class II molecules (H-2 locus). In F1 crosses, resistance was the dominant phenotype. All studies were conducted with female mice; male mice were more susceptible to infection. Immunization of the susceptible mice with a live, attenuated mutant protected them from infection, indicating that they could make a protective immune response to the organism. Backcross experiments suggested that a single locus might be the primary determinant of this phenotype [46]. In radiation chimera studies, resistance was conferred by the transplant of T-cells from the resistant strains. Other investigators reported that DBA/2 mice are also relatively resistant to intranasal infection while BALB/c mice are susceptible [47]. They found that the DBA/2 mice produced much more IFN-γ in their lungs, while susceptible mice produced more IL-4. Administration of IFN-γ to susceptible BALB/c mice improved their resistance to infection, whereas treatment of resistant mice with neutralizing anti-IFN-γ antibody increased their susceptibility, suggesting that these cytokines play a causative role in genetically determined resistance. There is also a study indicating that IL-12, a cytokine that stimulates a T_H_1 immune responses including IFN-γ, decreases the number of organisms in tissues after experimental infection [48].

Studies of cytokines in intraperitoneally infected genetically resistant (DBA/2) and susceptible (C57Bl/6) mice revealed that C57BL/6 mice made about 1000-fold more IL-10 and 10-fold more IL-4 in infected spleens and lungs than resistant DBA/2 mice after an I.P. infection [49]. In contrast, DBA/2 mice had more IL-12p40 in their lungs than did C57BL/6 mice. DBA/2 and C57Bl/6 mice made equivalent amounts of IFN-γ, IL-6, and IL-2. Most importantly, C57BL/6 mice with a deletion of the IL-10 gene were more resistant to infection than control mice and were equally resistant to infection as DBA/2 mice. C57BL/6 mice with an IL-4 deletion were somewhat more resistant to infection than control C57Bl/6 mice, but not as resistant as IL-10 deletion mice or DBA/2 mice. Therefore, IL-10 has a major influence on the course of infection in these mice.

Genetic mapping of the I.P. infection resistance phenotype was done in the BXD recombinant inbred mice available at that time [50]. Recombinant inbred mice are derived by intercrossing F1 animals (in this case C57BL/6 and DBA/2) for many generations to obtain inbred lines that are homozygous for different combinations of reassorted genes on all the non-sex chromosomes from both of the parental strains, which allows for the mapping of genes by comparison with previously mapped genes. In contrast to a previous study suggesting that a single gene determined resistance, the resistance phenotype was determined by at least two genes, one on chromosome 4 and the other on chromosome 6. The number of mapped genes was much smaller at the time than it is now, and there are many more RI BXD lines that could be tested, which could increase the likelihood of identifying more resistance loci/genes.

Dectin-1 plays a major role in innate resistance to murine *Coccidioides* infection and one difference between the C57BL/6 mice and the DBA/2 is a base-pair change in intron 3 of the *Clec7a* gene in C57/BL6 mice that leads to splicing out of the exon that encodes most of the stalk of Dectin-1, resulting in a truncated version of Dectin-1 (see Section 2.4.1). Humans also are known to alternatively splice *Clec7a*, resulting in the expression of at least two isoforms that can be expressed on cell membranes (see Section 2.6) [51].

**Figure 2:**
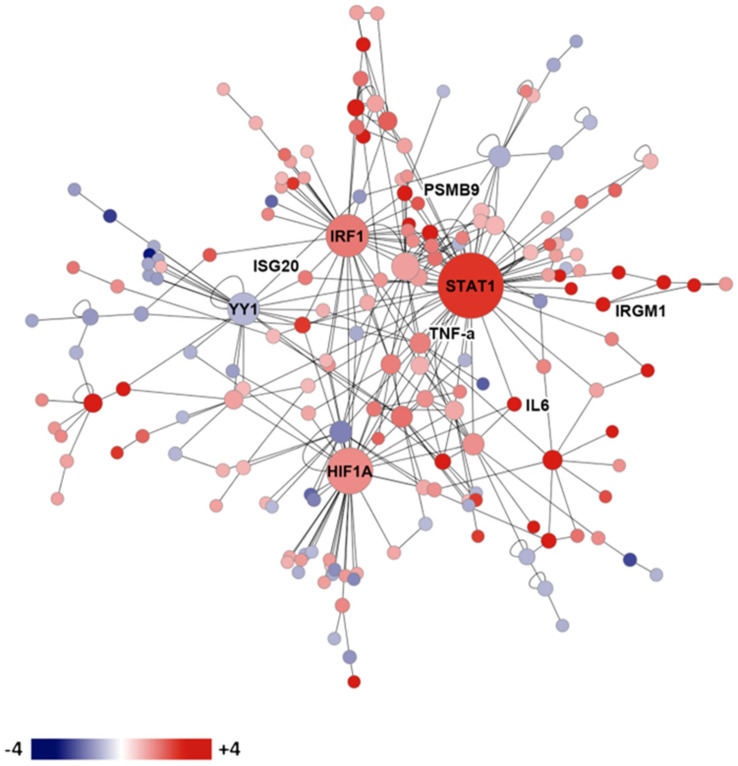
Gene expression network in infected genetically susceptible and resistant mice. Legend: protein expression network of gene transcription in resistant DBA/2 versus susceptible C57BL/6 mice on day 14 after infection. Red shading indicates more expression in DBA/2 than C57BL/6, while blue shading indicates the reverse.

The expression of mouse genes in the lungs of resistant and susceptible infected mice was compared using microarray technology [52]. Figure 2 shows the gene expression network resistance of DBA/2 mice versus susceptible (C57BL/6) mice on day 14 after intranasal infection. Many interferon-stimulated genes, cytokines, as well as histocompatibility genes were preferentially expressed in the genetically resistant mouse strain. Upregulated transcription factors in the resistant mouse strain included hypoxia inducible factor 1, interferon regulatory factor 1, and STAT1. These results indicate that a wide variety of genes are differentially expressed in the lungs of susceptible and resistant mice after infection, but they do not indicate which, if any, of these differences are most important for resistance, nor what cells are using those transcription factors to produce the cytokines [52].

### 2.6. Genetic Predisposition to Severe Disease in Human Beings

A small number of people with disseminated coccidioidomycosis have been found to have Mendelian mutations in the IL-12/IFN-γ pathway [53,54], evidence for the importance of IFN-γ and CD4 T_H_1 cells in immunity to coccidioidomycosis. A gain-of-function mutation in the STAT1 IFN-γ/IL-12 signaling protein that dysregulates signaling has also been observed in two patients with disseminated disease [55]. Recently, a genomic study of 58 patients with disseminated coccidioidomycosis found variants in genes coding for the IL-12/IFN-γ-signaling pathway, the innate immune-signaling pathway and the NF-κB- and IL-17-signaling pathways [6]. One variant found in a family with three generations of disseminated coccidioidomycosis was a missense mutation in STAT4 [36]. To further investigate this variant, this mutation was introduced into the mouse genome and resistance to infection was evaluated in mice infected with *C. posadasii* strain 1038 (a lower virulence clinical isolate that causes indolent infection in mice) [56]. The mutant mice became moribund much more quickly than the controls, indicating that this missense mutation has a dominant negative effect on the course of the disease. By contrast, C57BL/6 mice with only one1 STAT4 gene completely knocked out survived as long as the controls, which indicates that the E626G point mutation in STAT4 is dominant negative and suggests that only a single copy of the gene is required to have an observed effect on susceptibility to severe coccidioidomycosis in humans. [56] As expected, T-lymphocytes from C57BL/6 Stat4^E626G/+^ mice made less IFN-γ in response to IL-12 and IL-18. The number of T-lymphocytes in the mediastinal lymph nodes was also lower in the infected mutant mice than in the controls. 

All these mutations have a profound effect on T_H_1 or T_H_17 immunity, which are critical host responses. People who have low numbers of T-cells or poor T-cell function because of infections, such as HIV/AIDS, immunosuppressive malignancies, or immunosuppressive medical therapies, are also at a high risk for severe infection and/or disseminated infection [2,57,58].

There is also a marked association with ethnicity with the course of human infection. African Americans are significantly more likely to develop disseminated and/or lethal disease than are those of European descent [59,60,61,62,63]. Filipinos and Filipino Americans are also much more likely to develop disseminated disease than people of European descent [61]. There are some suggestions in the literature that Hispanics are more likely to develop severe or disseminated disease, but most experts think this is unproven. The risk of infection seems to be the same in African Americans and people of European descent, and although it is difficult to exclude sociological and environmental factors, the difference in susceptibility to dissemination rather than infection has been seen in many studies in the military and in prisons, where one would expect nutrition and housing to be similar for all those exposed.

One study has correlated the ABO blood groups and some HLA alleles with the severity of infection in three broad ethic groups: Caucasians, African Americans, and Hispanics [64]. There was evidence of a linkage of severity of disease to blood group antigens only in the Hispanic patients. However, HLA DQB1 alleles were associated with decreased or increased risk in all three populations.

**Figure 3:**
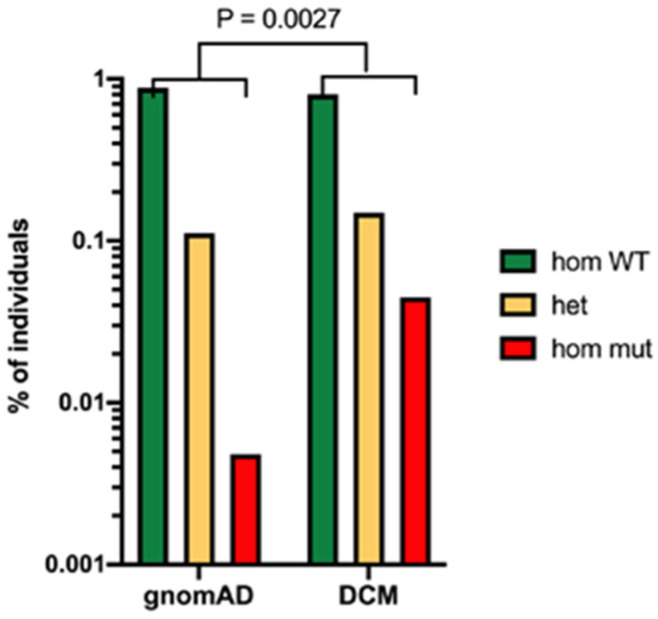
Evidence for the importance of Dectin-1 polymorphism in risk of disseminated coccidioidomycosis. Legend: Frequency of the *clec7a* mutation in patients with disseminated coccidioidomycosis (DCM) compared to the total population (estimated by gnomeAD). *p* = 0.0027 Fisher’s exact test [13].

A recent study has conducted extensive genetic analysis of people with disseminated coccidioidomycosis using whole-exome sequencing [13]. This approach makes no assumptions about the polymorphisms that might be associated with disseminated disease. They studied 67 patients in an exploratory set and 111 patients in a validation set. In the exploratory set, 2 patients had STAT3 mutations and 34 had defects in the β-glucan sensing pathway. These mutations included point mutations in the *clec7a* gene coding for Dectin-1 and a PLCG2 variant coding for phospholipase C γ-2, which is activated via Dectin-1 (Figure 3).

Mutations in DUOX1 or DUOX1A were also more common in patients with disseminated coccidioidomycosis than controls. DUOX1 and its obligate accessory maturation factor, DUOXA1, are expressed on the apical surface of specific epithelial cells and DUOX1 releases H_2_O_2_ in response to calcium-mobilizing agonists [65]. The DUOX mutants did not produce as much H_2_O_2_ in response to Dectin-1 agonists as controls [13].

### 2.7. Effector Mechanisms

How the host kills or inhibits growth of the fungus has yet to be established. Spherules are largely extra-cellular, but small round cells and endospores are small enough to be ingested by PMN, DC, and macrophages, thus both intra-cellular and extra-cellular killing can occur. The role of the reactive oxidative synthase (ROS) or oxidative burst in experimental murine infection was explored by comparing the course of infection in C57BL/6 NADPH gp91phox^−/−^ mice to controls [14]. These mice are a mouse model of human chronic granulomatous disease (CGD). CGD mice were not more susceptible to pulmonary infection (as measured by CFU) at a relatively low inoculum, and only slightly more susceptible to infection at a higher inoculum. Immunization rendered CGD mice completely resistant. CGD mice made a more vigorous inflammatory response than the controls, and most of the inflammation was due to PMNs. The infected CGD mice also made more TNF-α, IFN-γ, and IL-17 than the controls. In addition, arthroconidia were much more resistant to H_2_O_2_ in vitro than *Aspergillus fumigatus* spores; spherules and arthroconidia did not differ in susceptibility to this oxidative stress. These experiments suggest that the oxidative burst is not required for innate or acquired immunity to coccidioidomycosis. In support of that observation, there are no reports of disseminated coccidioidomycosis in patients with CGD [66]. However, the NADPH gp91phox^−/−^ mice are on a C57BL/6 background and even control C57BL/6 mice are very susceptible to infection. Deletions in a more resistant mouse strain might yield a different result.

Experiments by another group using the same C57BL/6 gp91phox^−/−^ mice found similar but not identical results [67]. In those experiments, C57BL/6 gp91phox^−/−^ mice died more quickly than controls after challenge with a *C. posadasii* isolate but the number of organisms recovered from the lungs was identical. Once again, immunization protected both wildtype and mutant mouse strains. The infected CGD mice made more pro-inflammatory cytokines than the controls. Differences in virulence of the two fungal strains may be a factor in the different results of these two studies.

The role of inducible nitric oxide synthase (iNOS) has also been investigated in C57Bl/6 iNOS^−/−^ mice [68]. The survival curves of the wildtype and iNOS deletion mice were identical and the number of organisms in the lungs were similar at 7 and 11 days after infection, although the iNOS^−/−^ mice had a slightly higher number of CFU in the spleen than the controls. Both normal and iNOS^−/−^ animals responded to immunization. There was also no difference in the expression of cytokines in normal and iNOS-/- mice. However, the iNOS^−/−^ mice are on a C57BL/6 background and these mice make much less NO after infection than DBA/2 mice [69]; therefore, the iNOS^−/−^ results cannot exclude the importance of iNOS in hosts that produce more iNOS in response to this infection.

## 3. Acquired Immunity

In immunocompetent people, a self-limited primary coccidioidomycosis infection protects against a second infection in the vast majority of cases [1]. This observation has prompted the search for an understanding of the mechanisms behind this protective immunity and attempts at vaccine development. Despite the agreement that T-cell mediated immunity is required for resistance to infection, the detailed cellular mechanisms for the control of infection are still elusive. This is clearly one of the largest knowledge gaps in our understanding of immunity to *Coccidioides*.

It was recognized very early that a positive skin test (a T-cell mediated immune response) and a negative or low complement-fixing (CF) antibody response was associated with self-limited infection in people [1,70]. In addition, since an elevated CF antibody titer is associated with a poor outcome, it was thought that antibody responses were not protective. The first data in experimental animals about the mechanisms of protective immunity showed that T-lymphocytes were important for immunity. Mice lacking T-lymphocytes (athymic nude mice) were more susceptible to infection than controls [71] and immunity was transferred primarily by spleen cells enriched for T-lymphocytes [72].

### 3.1. B-lymphocytes

Because of those transfer experiments, the authors suggested that B-cells were not important for the transfer of resistance, but that the cell fraction methods were crude at that time. It has been reported that serum antibodies do not transfer immunity from immune mice to recipients [73]. Subsequent studies on the role of B-cells have been contradictory. One found that B-cell deficient mice could not be immunized to the same extent as intact mice [74]. Another study found that B-cell deficient mice were no more susceptible to infection than controls (as measured by quantitative culture) and could be fully immunized [75]. The B-cell deficiencies in those experiments were caused by different mutations and the immunizing antigens were different, which may explain those discrepancies. However, a review of the literature does not reveal reports of severe coccidioidomycosis in humans with isolated hypogammaglobulinemia or primary B-cell deficiencies, indicating that these cells probably do not play a crucial role in the control of human coccidioidomycosis in the way that T-cells do.

### 3.2. T-Lymphocytes

#### 3.2.1. Studies in Mice

Although there was general agreement that T-lymphocytes were required for acquired immunity, the next question was what type of T-lymphocyte was protective. The T_H_1 T_H_2 paradigm was initially discovered in the 1980s by cloning CD4 T-cells and demonstrating that distinct clones made different cytokines [76]. CD4 T_H_1 T-lymphocytes produce pro-inflammatory cytokines, such as IFN-γ, IL-12 and chemokines, while T_H_2 CD4 T-cells produce anti-inflammatory cytokines such as IL-4 and IL-10. A T_H_1 T-cell response is important for the control of intracellular pathogens, while a T_H_2 is an important response to parasites and is also important for downregulating over-exuberant inflammation [77]. However, it is now clear that many more functional subsets exist that that play different roles in the immune response [77]. Nevertheless, there are a number of experiments addressing the T-lymphocyte subsets and cytokines involved in acquired immunity to coccidioidomycosis.

Adoptive transfer of cells from mice that were immunized with a live, attenuated mutant showed that T-cells lacking all antigen receptors could not transfer immunity but that CD4+ (helper type) T-cells could transfer immunity [75]. However, CD4-deficient mice were protected by immunization, which suggested that CD8 T-lymphocytes might play a role in immunity. Mice with a beta 2 microglobulin or a perforin deletion could be successfully immunized, suggesting that cytotoxic T-cells were dispensable for immunity. In addition to defining the types of T-cells required for acquired immunity, this study also showed that TNF-α, but not IFN-γ, was required for acquired immunity. Other experiments using immunization with a recombinant protein yielded somewhat different results [78]. MHC class II deletion mice lack all helper T-cell function, and were not protected by immunization, but both CD4- and CD8-deficient mice were protected by immunization. In this study, both IL-12 deletion and IFN-γ deletion mice were not protected by immunization. Although the IFN-γ results are different in these two reports [75], the immunization was conducted using different antigens.

**Figure 4:**
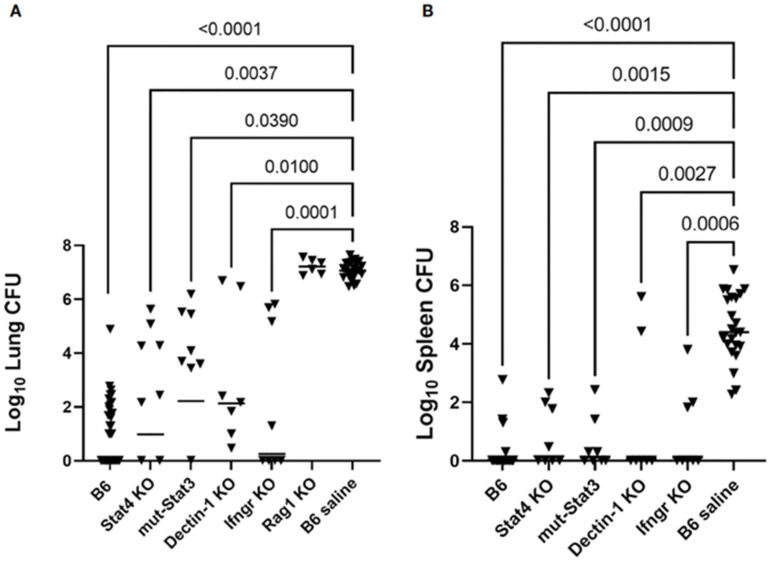
Immunization of mice with targeted deletions in the immune response. Legend: Mice with the indicated deletions were immunized with the Δ*cps1* strain: black triangles—CFU. (**A**) CFU/lung and (**B**) CFU/spleen [36].

A recent study has evaluated the ability to immunize and protect mice with targeted deletions to the immune system with the Δ*cps1* mutant (see Section 4.2) [36]. Figure 4 shows that mice lacking Stat4, Stat3, Dectin-1, or the IFN-γ receptor could be immunized, as indicated by reduced CFU in the lungs and spleens. In contrast, Rag1 knockout mice, lacking all lymphocytes, could not be immunized. These experiments indicate that immunization with this live, attenuated mutant is effective in mice with several different types of immunodeficiencies. It seems to indicate that multiple pathways can lead to protection when a live, whole-cell vaccine is given to the mice, except that the requirement for T-cells is absolute. While there is abundant evidence that people with inherent acquired immunodeficiencies are prone to worse disease, vaccination with a broad array of antigens could potentially reduce disease severity. These data support the safety and efficacy of a potential live, attenuated vaccine, even in immunocompromised people.

Other studies that used a live, attenuated *cts2/cts3* mutant (ΔT) to immunize mice found that T_H_17 cells are critical for adaptive immunity [79,80]. Vaccination was protective (as measured by quantitative culture) in wildtype mice but not in IL-17RA^−/−^ mice; IL-17A^−/−^ mice were only partially protected. Immunization of the wildtype mice increased the number of IL-17+ T-cells in the lung and IL-17 secretion by splenocytes in vitro. A follow-up study reported that CD4-IFN-γ (T_H_1), CD4-IL-5 (T_H_2) and CD4-IL-17A (T_H_17) T-cells were present in the lungs of immune mice after challenge, indicating that all three types of T-cell immunity are elicited by this live vaccine [81]. This study also showed that IL-17A^−/−^ mice were successfully vaccinated, while IL-17RA^−/−^ mice were only partially protected by vaccination. These studies also emphasize the importance of the T_H_17 lymphocytes. To our knowledge, studies of invariant T cells and NK cells have not been conducted.

#### 3.2.2. Immunity in Humans

Most people infected with *Coccidioides* spp. have no symptoms, or a self-limited pneumonia, and develop a positive skin test, which is an indicator of T-cell mediated immunity. Smith reported that a positive skin test with a complex antigen from cultured *C. immitis* was associated with a much better clinical outcome than a negative skin test [82]. However, the predictive importance of a positive skin test in the era of effective antifungal therapy has been controversial. One study found that the skin-test status at initial presentation did not predict relapse after cessation of antifungal treatment, but that repeated positive skin tests before the completion of antifungal therapy were associated with a good outcome [83]. Another study also found that a negative skin test at presentation was not associated with recurrence of disease after therapy was stopped [84]. Almost all patients in this study were treated with antifungal drugs and fewer than 1% of the patients in either the skin-test-positive or skin-test-negative groups relapsed. More comprehensive studies determining the correlation between skin-test reactivity over time and clinical outcome are warranted.

A number of studies found that those people had a positive lymphocyte proliferation response to *Coccidioides* spp. antigens in vitro [85,86,87]. At the time, lymphocyte proliferation was accepted as an in vitro test of T-cell-mediated immunity; enrichment of T-cells was difficult and not often done. Ampel and colleagues reported that people with a positive skin test produced more IL-2 and IFN-γ than controls in response to *Coccidioides* spp. Antigens [88]. Antigen stimulation of lymphocytes from immune individuals also resulted in increased expression of CD69, a marker of T-lymphocyte activation, which conclusively demonstrated that T-lymphocytes were being activated [89]. A study in a small number of subjects found that healthy people who were immune and or had disease limited to the lungs made more IFN-γ and IL-12 in vitro than uninfected controls or patients with disseminated disease in response to spherule antigens, suggesting that a T_H_1 immune response was associated with a good outcome [90].

Antigen-stimulated T cells from healthy immune subjects produced more IFN-γ than T-cells from patients with disseminated disease or active chronic pulmonary disease, further evidence that a T_H_1 immune response is associated with a good outcome [91]. This study was also limited because of its small size. The types of cytokines made in response to antigen stimulation in vitro by circulating lymphocytes from patients with primary pulmonary coccidioidomycosis have been reported [92]. The primary cytokines produced were IL-2, IFN-γ, GM-CSF, IL-1RA, IL-1β, IL-13, and TNF-α. Except for IL-RA, all these cytokines were produced at higher levels by patients with primary disease than healthy immune skin-test-positive controls. Patients who produced more IL-2 in response to antigen stimulation were less likely to require anti-fungal therapy. More extensive studies comparing immune subjects to patients with active disease are clearly needed.

The Involvement of Tregs in coccidioidomycosis was investigated by Hoyer and colleagues, who found that pediatric patients with persistent disease had higher numbers of Treg cells in their peripheral circulation than those with resolved disease or healthy controls, suggesting that the Treg cells might be interfering with the development of a protective immune response [93]. In contrast, a study of granulomas in patients who resolved their pneumonias but had residual granulomas did not find many Treg cells, and those that were present were not associated with IL-10 [94]. More studies of Treg cells in this disease are needed.

The importance of the T_H_1:T_H_2 ratio in the control of disseminated coccidioidomycosis has been suggested [95] in a report of a young child with severe disseminated coccidioidomycosis involving the skull and multiple areas of the spine. He did not respond to antifungal treatment with two active antifungal drugs. The results of a skin test were not reported. He was HIV-negative and an extensive evaluation for genetically determined immunodeficiency did not reveal a monogenic cause for his disease susceptibility. However, stimulation of his T lymphocytes with a crude *Coccidioides* antigen revealed a T_H_1:T_H_2 ratio of 0.4, which is considerably lower than was expected [95]. An evaluation of transcript variants by RNA sequencing revealed the predominance of a short transcript of the *IL12RB1* gene, which is nonfunctional, and explained both his cells’ poor responses to IL-12 stimulation and his consequently poor development of type 1 immunity. Because his disease was progressing, treatment with IFN-γ was initiated, in addition to antifungal drugs. This led to improved transcription of the *IL12RB1* gene with full-length transcript and partial restoration of IL-12 signaling, but his clinical disease did not improve sufficiently. His stimulated T cells made high levels of IL-4, so he was also treated with dupilumab, a monoclonal antibody that blocks IL-4 and IL-13 receptor activities. His T cells showed normalization of the T_H_1:T_H_2 ratio. Treatment with antifungal drugs, surgical debridement, and these two biologic agents led to a dramatic clinical improvement.

**Figure 5:**
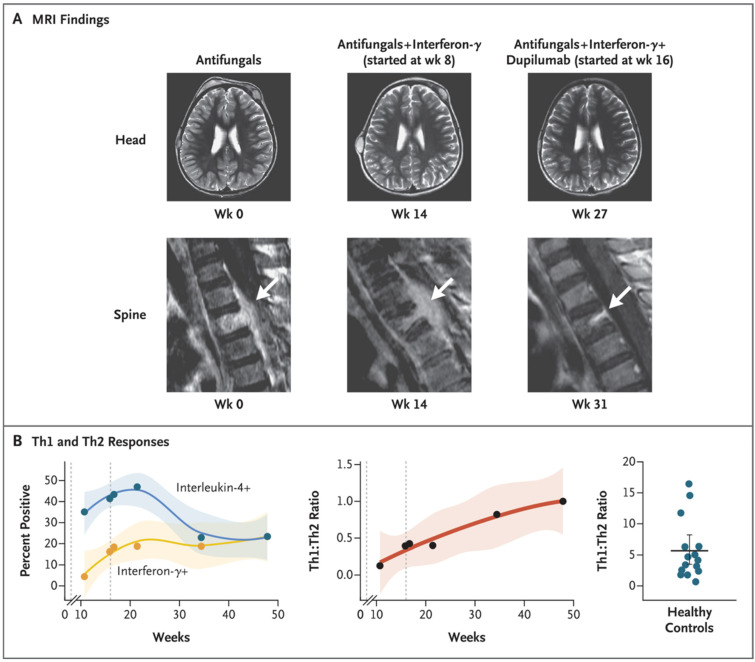
Clinical and laboratory findings in a child with disseminated disease. Legend: Panel (**A**) shows magnetic resonance imaging (MRI) of the head and spine at baseline and during treatment. The arrow indicates inflammation. Panel (**B**) shows the percentage of CD4+ T cells producing interferon-γ (T_H_1 cells) or interleukin-4 (type 2 helper T [T_H_2] cells) (**left**) and their ratio (**center**) over time. The first dashed line represents the initiation of interferon-γ treatment, and the second dashed line represents the initiation of dupilumab treatment. Shading indicates the 95% confidence interval. The Th1:Th2 ratio for 15 healthy controls is shown on the right. From [95]; reproduced with permission.

Figure 5 shows the change in T_H_1:T_H_2 ratio and his clinical improvement. This case study suggests that, at least in this patient, an imbalance of T_H_1:T_H_2 T-lymphocyte functions can cause severe disease. Whether these findings will be applicable to a larger group of patients with severe disseminated coccidioidomycosis requires further investigation.

One limitation of most of the immunological profiles in both mice and humans is the poorly defined and standardized antigens that are used for in vitro studies of immune cells. Almost all experiments have been conducted with a toluene lysate of spherules or a product that is made by the mechanical disruption of spherules known as T27K [96,97]. T27K contains both protein and carbohydrate; analysis by SDS-PAGE showed that the products migrate as a diffuse band with molecular weights ranging from 20–140 kDa [98]. Repeated immunization of mice with 200–400 microgram of T27K with alum as an adjuvant protects mice from a lethal challenge. It is not clear which components of T27K elicit protective immunity, although the most likely candidates are proteins or glycoproteins. In the absence of this information, it is very difficult to standardize these antigen preparations.

All these human studies suggest that a T_H_1 immune response is associated with a good outcome. However, extensive studies of antigen-specific T-cell subsets or IL-17 T-cells have not been conducted in immune people or patients with active infection. Further studies of the immunology of coccidioidomycosis in human beings are sorely needed.

## 4. Vaccines

### 4.1. Whole Spherule and Spherule Extract Vaccines

Because human infection almost always conferred life-long immunity, the search for an effective vaccine was initiated long before the era of modern immunology. Some of the earliest studies showed that spherules were more effective *Coccidioides* vaccines in animals than arthroconidia [99]. Immunization of mice with a very large amount (2 mg) of formalin-fixed spherules (FKS) or endospores prevented mortality from a high-dose pulmonary challenge. Smaller amounts of FKS were less protective as were arthroconidia or mycelia. This finding has been confirmed repeatedly, although enhanced innate immunity to glucan may play a large part in conferring protection, especially since very large doses were administered repeatedly [73]. Later studies with the poorly characterized T27K (an extract from *Coccidioides*) vaccine showed that 1 mg doses given three times with alum were also protective against a large pulmonary challenge [96].

A phase-3 randomized double-blind human vaccine trial of a formalin-killed spherule vaccine was conducted based on animal protection studies. This study enrolled 2867 skin-test-negative participants in the San Joaquin Valley [100,101] who were immunized three times with a large dose (1.75 mg) of formalin-killed spherules and compared to unimmunized controls. The total number of cases was low; only 12 confirmed cases and 13 suspected cases in the controls compared to 9 confirmed cases and 9 suspected cases in the immunized group, with all cases being mild. The difference in the number of infections between groups did not achieve statistical significance. No immunologic studies were reported. The vaccine injection site side effects were severe, and this approach has not been pursued.

### 4.2. Live Attenuated Mutants

Several live, attenuated mutants are highly active vaccines in experimental infections. The first ones were made by random mutagenesis of the *C. immitis* RS strain by UV irradiation [102]. One of these, 95–271, was temperature sensitive and auxotrophic for para-aminobenzoic acid and riboflavin. This mutant was non-virulent and was effective as a live vaccine [34,75,102].

A genetically defined mutant was engineered by deleting two chitinase genes (CTS2 and CTS3) and the ARD1 gene in *C. posadasii* C735 [79]. This mutant grows normally in the mycelial phase but forms sterile spherules that do not produce endospores [79]. The strain (referred to as ΔT) is a highly effective vaccine in mice [79,81]. Two different strains of susceptible inbred strains of mice that were immunized with ΔT and then challenged intranasally with 7.5 × 10^4^ live arthroconidia (a very large inoculum) were protected. The surviving BALB/c mice had much lower numbers of organisms in their lungs than the controls, and C57BL/6 lungs were sterile. This live, attenuated vaccine is also evaluated using human HLA-DR4 transgenic mice that the vaccinated mice show reduced fungal burden and prolong survival compared to the control mice [103].

As was mentioned earlier, another engineered mutant was made by deleting the *CPS1* gene [104]. The Δ*cps1* mutant, grew slower than the wildtype in the mycelial phase and produced only 10% as many arthroconidia as the parent strain. The mutant formed spherules in spherule-inducing culture conditions, but they were significantly smaller than the wildtype spherules and the majority degraded between 48–72 h of cultivation without producing endospores. The mutant was completely avirulent in genetically susceptible but immunocompetent mice, even when an inoculum of 4400 arthroconidia was used. When highly immunocompromised mice were infected with the mutant, all but one animal was sterile at necropsy, and it had only minimal fungal growth. Immunizing C57BL/6 or BALB/c strains of susceptible mice with 10,000–50,000 Δcps1 arthroconidia intraperitoneally, intranasally, or subcutaneously was equally protective against a lethal challenge and resulted in much lower fungal burden in the lungs. The immunized and infected mice did have significant fungal growth in lungs, but minimal or no dissemination to the spleen. However, a subsequent study demonstrated 100% survival of immunized mice 6 months after challenge with *C. immitis* or *C. posadasii* and 40% of the mice had no detectable fungal burden at sacrifice; thus, immunization with the Δcps1mutant was remarkably robust and durable. Killed Δ*cps1* arthroconidia did not protect mice, indicating that the highly protective immunity is most likely related to the fact this organism presents antigens associated with the dimorphic switch and at least rudimentary attempts to make endospores [105].

This vaccine has also been tested in dogs in a pulmonary challenge model [106]. As expected, viable Δ*cps1* were not recovered from subcutaneous tissues at the vaccination sites or from draining lymph nodes 42 days after immunization with 10^4^–10^5^ arthroconidia, except for a single primary injection site that yielded a minimal growth. Two doses of 10^4^–10^5^ viable arthroconidia given 28 days apart conferred significant protection to the animals against a nebulized aerosol challenge of 10^4^ *C. posadasii* Silveira arthroconidia, as measured by quantitative culture of the lung at necropsy (56 days after infection) and a disease score that included serial thoracic radiography, clinical pathology, lung and thoracic lymph node histopathology, and serology in addition to lung and lymph node fungal burdens. Although it is not known with certainty, it is believed that this challenge is much larger than that which occurs in naturally infected dogs. The infection of pets, especially dogs, is an important veterinary problem in endemic areas, and this might be a useful animal vaccine. It is under development as a vaccine for dogs.

The mutant of a WOPR family transcription factor, Ryp1, was also generated in *C. posadasii* Silveira [107]. Ryp1 is a transcription factor that is a master regulator of yeast formation in *Histoplasma* [108]. The *C. posadasii* Δ*ryp1* mutant has severe defects in morphology and does not form spherules. It is avirulent in a mice model of infection, but is not an effective live vaccine [107]. The reason for this is not clear, although the inability to differentiate into spherules is an attractive hypothesis.

It is clear that several live, attenuated mutants capable of forming spherules are very active vaccines. One potential issue is difficulty in mass producing and storage of viable vaccines. Other major concerns with this approach are the potential risk of infection in immunocompromised human hosts and acceptability of immunization with a live vaccine by the public. This is particularly true since most cases of coccidioidomycosis are self-limited. Whether or not these mutants are realistic candidates for a human vaccine is unclear. However, they are certainly useful tools for better understanding the protective immunity.

### 4.3. Vaccine Candidate Proteins

Sequencing the genome of *Coccidioides* spp. allowed for the discovery of thousands of predicted proteins as potential vaccines. Some, such as those that were highly conserved in both fungi and mammals, could be quickly eliminated, but this still left a daunting number of candidate proteins. However, not all proteins, even those expressed on the surface of the spherule are effective vaccines. For example, spherule outer wall glycoprotein is highly expressed on the spherule surface and is highly immunogenic as measured by antibody and T-cell responses, but it is not protective [109]. Dozens of other antigenic proteins have been found to lack protective activity despite eliciting an immune response. Unfortunately, there is currently no way to predict which spherule proteins will elicit protective immunity. One attractive candidate is the set of genes expressed in Δ*cps1* but not in Δ*ryp1* in vivo. Future studies focusing on comparisons of these two mutants and others that are defective in spherule formation will be valuable in defining *Coccidioides* vaccine antigens.

Although an antigen known as Ag2/PRA is not located on the surface of the spherule, it was found to be an effective protein and DNA vaccine against an intranasal challenge [110]. There have been at least several dozen published experiments showing that vaccination with Ag2/PRA is protective in C57BL6 mice challenged with 10–50 arthroconidia [78,111,112]. Nevertheless, other investigators have found that Ag2/PRA recombinant protein was not an effective vaccine against a pulmonary challenge, although a cDNA vaccine was effective, especially when it included a cDNA coding for IL-12 [113,114]. These disparities between the results from different laboratories may be due to methodological differences, such as immunization dose and schedule, adjuvant, size of infecting inoculum, virulence of the challenge strain, or differences in the susceptibility of the mouse strains.

In most experiments described in Table 1, mice were immunized with relatively low doses of antigen (1–10 μg) and boosted 4 weeks later. After another 4 weeks they are challenged with about 10–50 arthroconidia intranasally. Necropsy at day 14 after infection and quantitative culture was a typical measure of efficacy. In many, but not all instances, both survival studies and quantitative cultures were done. It subsequently became clear that some of the discrepancies in results were because *C. posadasii* Silveira is a more virulent strain than *C. immitis* RS and it is more difficult to protect BALB/c mice than C57BL/6 mice.

Calnexin is a chaperone found in the endoplasmic reticulum and on the surface of fungi. It was found to be an effective vaccine for *C. posadasii* and *Blastomyces dermatitidis* [115]. Three immunizations with 10 μg of calnexin in glucan particles resulted in a 100-fold decrease in the number of *C. posadasii* recovered from the lungs and spleens of infected mice. Survival studies were not reported, and this approach has not been pursued. Immunization with a Triton-114 extract of the *C. posadasii* cell wall is highly protective. An aspartyl protease was predicted by bioinformatic techniques to be a GPI-anchored protein localized to the cell wall [116]. That protein was also predicted to have five promiscuous T-cell epitopes, and so was tested as a vaccine. Immunization with the recombinant aspartyl protease protein significantly decreased the number of organisms recovered from the lungs and increased the survival of infected mice [116].

Several combinations of antigens were better vaccines than individual proteins. *Coccidioides*-specific antigen (CSA) is only modestly protective as measured by survival [112]. However, the combination of CSA and Ag2/PRA is significantly more effective than either antigen alone. This is true whether the immunization is conducted with the two individual proteins or a fusion protein [112]. This idea has been pursued with other combinations. One approach evaluated proteins from a TX-114 extract of spherule cell walls for reactivity with human immune serum. Some of the immunoreactive SDS-PAGE bands were identified by proteomic techniques, and the epitopes predicted to bind to many human MHC II proteins were chosen for further evaluation [117]. Predicted epitopes from three proteins were made into a recombinant protein and tested for activity in HLA-DR4 transgenic C57BL/6 mice. Immunization of the transgenic mice with this protein using CpG oligonucleotides as an adjuvant [118] resulted in a significant decrease in the number of organisms recovered from the lung, but had minimal effects on survival. Using glucan as an adjuvant, immunization elicited a robust IFN-γ and T_H_17 immune response, and had a significant, but less dramatic effect on the numbers of organisms in the lungs and spleen and survival. HLA-DR4 transgenic C57BL/6 mice were used for these experiments in an attempt to mimic the human immune response more closely; the effect of vaccination in normal C57BL/6 mice was not reported.

A fusion protein containing eight active components was constructed by Hung and colleagues [119]. A cDNA containing the components (Ag2/PRA, CSA, immunoreactive peroxisomal matrix protein, and the five-epitope protein described above) was constructed and recombinant protein was produced. This protein, referred to as rCpa1, was loaded into a variety of glucan particles which were compared as adjuvants. rCpa1 elicited a vigorous T_H_17 immune response, especially when glucan–chitin particles are used as an adjuvant. Immunization with rCPA1 in glucan-chitin particles resulted in more than 100-fold decrease in organisms/lung. Glucan particles, glucan–mannan particles, and glucan–chitin–mannan particles were substantially less effective as adjuvants. rCpa1 in glucan–chitin particles protected C57BL/6 mice from lethal infection almost completely and HLA-DR4 mice were also protected from lethal infection, although much less well than wildtype animals. This combination appears to be the most active recombinant protein vaccine evaluated so far.

**Table 1:** Vaccine candidates evaluated in mice. Legend: ^1^ three immunizations of 1 mg; ^2^ Coccidioides-specific antigen, ^3^ monophosphoryl lipid A; and ^4^ recombinant protein with eight components. This table is adapted from [120].

Antigen	Form	Adjuvant	Activity	Reference
Live attenuated mutants	N/A	N/A	4 +	[79] [104]
Formalin-killed spherules	N/A	N/A	4 + mice - humans	[96] [101]
Spherule extract ^1^	N/A	Various	4 +	[96]
Ag2/PRA	Protein, DNA	Various	2 +	[110]
Ag2/PRA	Protein, DNA	Various	- protein 1 + cDNA	[113,114]
Expression library 1	DNA	None	3 +	[121]
Prp2	Protein	CpG	1 +	[122]
B-glucanosltransferase	Protein	CpG	2 +	[123]
Calnexin	Protein	Glucan and Adjuplex	2 +	[115]
Aspartyl protease	Protein	CpG	3 +	[116]
CSA ^2^	Protein	CpG and MPLA ^3^	1 +	[112]
Multivalent antigens				
Ag2/PRA CSA fusion protein	Protein	CpG and MPLA	3 +	[112]
Phospholipase, α-mannosidase and aspartyl protease peptide fusion protein	Protein	CpG glucan particles	2 +	[117]
rCpa1 ^4^	Protein	Glucan chitin particles	4 +	[119]

The type of adjuvant used with a subunit vaccine can clearly be critical for eliciting protective immune responses. Most of the recombinant vaccine studies were conducted with monophosphoryl lipid A and CpG oligodeoxynucleotides (CpG-ODN) or CpG-ODN alone as an adjuvant, but glucan–chitin particles also are clearly effective. Adjuvants are best understood in terms of the pattern-recognition receptor that they activate. With the current understanding of vaccine immunity, more rational choices can now be made [124]. Many new adjuvants have been developed recently although developing effective vaccine/adjuvant combinations with minimal toxicity remains a major challenge [125]. Comparisons of protein combinations to each other and comparisons of different adjuvants with protein(s) are needed to determine the most effective vaccine adjuvant combination. As seen above, a glucan or glucan–chitin particle adjuvant can make at least two protein vaccines much more active than CpG.

The testing of potential vaccines in a larger animal model of coccidioidomycosis is also important for pre-clinical development. The successful vaccination of dogs with the Δ*cps1* mutant is an important validation of the mouse vaccination experiments. Since one live, attenuated vaccine has been effective in dogs, experiments with protein vaccines in these animals might be useful.

It is difficult to know how to take a potential vaccine from the research lab to human trials, especially since some candidates elicited very strong immune responses but were provided with only modest protection. Either our understanding of the immunology is incomplete, which is certainly true, or the model that is being used to evaluate vaccine efficacy is not the best, or both. This area of research certainly deserves more study.

However, since the goal of these studies is to develop a human vaccine that is clinically and commercially viable, interest and input from pharmaceutical companies is really needed. It is not at all clear that the market for a Valley Fever vaccine is large enough to attract corporate support. Finally, whether the people in the endemic area will accept a vaccine is unclear at this point. Although there has been interest in the past, the experience with the COVID-19 vaccine suggests that acceptance might be a major problem.

## 5. Conclusions

The amount of research and clinical interest in *Coccidioides* spp., as measured by papers indexed in PubMed, has increased steadily and dramatically over the past 50 years, as has funding from the National Institutes of Health. However, in our opinion, the quality of the research and the potential for improving our understanding of the host response to the organism has increased even more dramatically. Very sophisticated techniques are currently employed to understand the innate and acquired immune responses to these fungal pathogens. Many of the experiments that are currently being conducted to understand the host responses would have been unimaginable ten years ago. Nevertheless, the older literature also contains some insights that should not be lost.

Understanding of the innate and acquired immune response is clearly a work in progress since many aspects of those host responses have not been evaluated, especially in human beings. This review does contain some notes of caution. For example, it is clear from several studies that experiments with a single strain of *C. immitis* or *C. posadasii* might not apply to all strains. Although the majority of studies have been done in mice, understanding the human host responses to infection is the ultimate goal and many more human studies are needed. Understanding the immune response in different groups of patients requires large sample sizes and careful clinical categorization, and a tool for that has recently been published [126]. Vaccine studies, in particular, illustrate that we need to be cautious about the strengths and limitations of our models and the importance of different types of immune responses.

Nevertheless, it is our opinion that the current state of *Coccidioides* spp. research is healthy and the future of coccidioidomycosis research is bright indeed. The amount of funding for research is increasing and the number of young investigators coming into this field is increasing steadily. These factors should increase our ability to understand the organism and the host responses to infection.

## Data Availability

Data are contained within the article.
